# Genome-wide identification, structural and gene expression analysis of *BTB* gene family in soybean

**DOI:** 10.1186/s12870-024-05365-1

**Published:** 2024-07-11

**Authors:** Hind Abdelmonim Elsanosi, Jianhua Zhang, Salma Mostafa, Xiaoyan Geng, Guisheng Zhou, Atef Hemaida Mohammed Awdelseid, Li Song

**Affiliations:** 1 Joint International Research Laboratory of Agriculture and Agri-Product Safety, Jiangsu Key Laboratory of Crop Genomics and Molecular Breeding, The Ministry of Education of China, Yangzhou University, Yangzhou, Jiangsu 225009 China; 2 Faculty of Agriculture, University of Khartoum, Khartoum, 11115 Sudan; 3 College of Horticulture and Plant Protection, Yangzhou University, Yangzhou, 225009 China

**Keywords:** Soybean, BTB domain, Abiotic stress, Nitrate response, Gene expression

## Abstract

**Background:**

The Bric-a-Brac/Tramtrack/Broad Complex (BTB) gene family plays essential roles in various biological processes in plants. These genes encode proteins that contain a conserved BTB domain, which is involved in protein-protein interactions and regulation of gene expression. However, there is no systematic reports on the *BTB* gene family in *G.max*.

**Results:**

In total, 122 soybean *BTB* genes were identified, which were classified into four groups based on the phylogenetic analysis. Gene structures analysis indicated that the number of exon–intron in *GmBTBs* ranges from 0 to18. Cis-element analysis revealed that most *GmBTB* genes contained cis-elements related to an abiotic stress response. In addition, qRT-PCR analyses indicated that most *GmBTBs* are significantly up-regulated under salinity, drought, and nitrate stresses. They suggested their potential for targeted improvement of soybean response to multiple abiotic stresses and nitrate availability.

**Conclusion:**

These results provide valuable information for identifying the members of the *GmBTB* gene family in soybean and could provide a functional characterization of *GmBTB* genes in further research.

**Supplementary Information:**

The online version contains supplementary material available at 10.1186/s12870-024-05365-1.

## Background

The *BTB* gene family, recently identified in plants, is characterized by the presence of the BTB domain, a highly conserved region spanning 120 amino acids and typically located at the N-terminus [1–3]. The core structure of the BTB domain comprises five α-helices and three β-strands [4]. The BTB domain enables the BTB proteins to form protein complexes and interact with other proteins, thereby modulating their functions [5, 6]. BTB proteins can be categorized into various subfamilies based on the additional function domain that can be combined with the BTB domain at either the N- or C-terminal, contributing to protein-specific functions and diversifying their roles in cells. These subfamilies include BTB-NPH3, MATH-BTB, BTB-BACK, BTB-ANK, BTB-only, BTB-TAZ, BTB-DUF, etc. [3, 7].

The BTB protein family has been identified in several plant species, revealing their involvement in various physiological and developmental processes, such as plant growth and development, hormone signaling, flowering time regulation, and stress responses [8]. Previous studies have reported the essential roles of *AtBTB2* and *AtBTB3* in regulating gametophyte development in *Arabidopsis* [9]. Additionally, *BT1* and *BT2* play significant roles in nitrate responses in both *Arabidopsis* and apple [10–12]. Studies have shown that overexpression of *AtBTB2* promotes lateral root development in *Arabidopsis* [10]. Interaction between *MdBT2* and *MdBHLH93* has been observed to regulate leaf senescence by degrading apple chlorophyll [13]. Furthermore, overexpression of *IbBT4* from sweet potatoes has significantly enhanced the drought tolerance in transgenic *Arabidopsis* plants [14]. The cucumber *BTB* gene is influenced by cold, drought, and salt stress, exhibiting varied patterns expression patterns in cucumber tissues [15]. Moreover, the expression of *AtNPR1* in tomatoes and wheat enhances the resilience of the tomato plant to bacterial and fungal infections, as well as boosts the defense response against *Fusarium head blight* (FHB) in wheat [16, 17].

Soybean (*Glycine max* (L.) Merr.) is a valuable crop that provides edible protein and oil for human consumption, along with being used in various industrial applications such as food processing to biofuel production [18]. With the ability to fix atmospheric nitrogen, soybeans play a vital role in sustainable agriculture by enhancing soil fertility and decreasing reliance on synthetic fertilizers [19]. As autotrophic sessile organisms, various environmental stresses, such as drought, salinity, and nutrition availability, often challenge them, which affects growth, development, geographical distribution, and application [20]. Soybean research and breeding programs focus on improving yield, disease resistance, and stress tolerance to enhance its productivity and adaptability to different regions. Genomic studies and genetic engineering techniques have facilitated the development of genetically modified soybean varieties, which possess traits such as stress resistance and enhanced nutritional profiles. For instance, overexpression of *BTB* genes in soybean has been shown increased resistance to *Phytophthora sojae*, but further research is needed to fully understand the role of these genes in stress responses [21].

To date, there has been a lack of comprehensive explorations of the BTB gene family in soybean. Therefore, this study aims to conduct a genome-wide investigation into the evolutionary characteristics and biological functions of *G.max* BTBs (*GmBTBs*). The analysis includes examining phylogenetic relationships, gene structures, chromosome localization, replication events, synteny, and promoter analysis. Furthermore, the expression patterns of *BTB* genes in different tissues were investigated using transcriptomics data. In addition, we screened some *GmBTB* genes that might be associated with abiotic stresses and nitrate response in soybean. Overall, this study comprehensively explores the *BTB* gene family in soybean and establishes a solid foundation for future functional analysis.

## Materials and methods

### Identification of *BTB* genes in the soybean genome

To identify soybean *BTB* gene family members, the protein sequences of *BTB* genes from *Arabidopsis* and rice were downloaded from the TAIR database (https://www.arabidopsis.org, accessed on 6 June 2023) and Phytozome (https://phytozome.jgi.doe.gov/pz/portal.html, v13.1, accessed on 6 June 2023), respectively. Different subfamilies of BTB proteins from *Arabidopsis* and rice were used as queries to search against the whole soybean genome by the BLASTP (http://blast.ncbi.nlm.nih.gov, accessed on 7 June 2023) program with an E-value < 1e-6. Furthermore, a search was performed in the Phytozome database using “BTB” or “POZ” as a keyword in the *Glycine max* reference genome (a4.v1 version). The retrieved sequences were used for domain searches in the Pfam (http://pfam.jouy.inra.fr, accessed on 9 June 2023) and SMART (http://smart.embl-heidelberg.de, accessed on 9 June 2023) databases with an E-value cutoff level of 1.0. Candidate genes lacking indispensable BTB domains were removed. Online server ExPASy (https://web.expasy.org/protparam, accessed on 10 June 2023) was utilized to analyze the number of amino acids (aa), theoretical isoelectric point (pi), and molecular weight (MW) of all GmBTB proteins. Plant-mPLoc server (http://www.csbio.sjtu.edu.cn/cgi-bin/PlantmPLoc.cgi, accessed on 12 June 2023) was used to identify the subcellular location of identified *GmBTB* genes.

### Sequence alignment and phylogenetic analysis

To classify *GmBTB* genes, the protein sequences of 122 *GmBTB*s were downloaded from Phtytozome v13.1 (accessed on 14 June 2023). Then, all sequences were aligned by MUSCLE in MEGA 11 [22]. The unrooted phylogenetic tree was constructed using MEGA 11 with the neighbor-joining (NJ) method using the p-distance method and bootstrap 1000 replication. The phylogenetic tree was organized using iTOL (https://itol.embl.de, accessed on 15 June 2023) [23].

### Chromosomal location, duplication, and genome synteny analysis

The TBtools software was utilized to build the chromosomal location picture of *GmBTB* genes based on gene physical position data. The One-Step Multiple Collinearity Scan toolkit (MCScanX) functions in TBtools software were used to perform gene duplication analyses, and the result was further visualized using Circos in TBtools (https://github.com/CJ-Chen/TBtools, accessed on 16 June 2023) [24]. Genes that were situated on the unassembled genomic scaffolds were eliminated from analyses. The syntenic analysis maps were constructed using the Dual Systeny plot among four different genomes (*Arabidopsis thaliana*, *Cicer arietinum*,* Oryza sativa*, and *Zea mays*).

### Ka/Ks analysis of *BTB* gene and domain

Non-synonymous (ka) and synonymous (ks) substitutions of each duplicated *BTB* gene were calculated using KaKs_calcu in TBtools (accessed on 16 June 2023). BTB domain protein sequences were retrieving from the SMART database (accessed on 24 April 2024). Subsequently, the corresponding coding sequence dataset was used to predict the Ka and Ks values in TBtools software.

### Gene structure and conserved motif

The *GmBTBs* gene structure was analyzed using TBtools software. Conserved motifs of *GmBTB* genes were analyzed using MEME (http://meme-suite.org/tools/meme, v5.3.3, accessed on 17 June 2023) [25]. The XML file containing motif pattern information obtained from MEME software was used to generate a motif distribution diagram by TBtools software [24].

### Cis-acting elements analyses

The upstream 1500 bp genomic DNA sequences of GmBTB proteins were downloaded from Phytozome v13.1 and submitted to PlantCARE (http://bioinformatics.psb.ugent.be/webtools/plantcare/html/, accessed on 19 June 2023) to survey the potential stress response and hormone-related cis-regulatory elements in the promoter region.

### Gene co-expression analysis and gene ontology annotation

The co-expression gene data for the soybean BTB proteins was acquired from the Phytozome database. A Gene Ontology (GO) enrichment analysis was conducted to gain insights into these genes’ functional characteristics. The analysis was carried out utilizing the SoyBase database (https://www.soybase.org/sbt, accessed on 25 June 2023). This approach allowed for identifying and exploring enriched GO terms associated with the soybean BTB proteins, shedding light on their potential biological roles and molecular functions.

### Gene expression pattern of the GmBTB proteins in soybean tissues

The expression patterns of *GmBTB* genes were investigated based on the FPKM values extracted from Phytozome (accessed on 27 June 2023), including eight tissues (root, root tip, lateral root, stem, leaf, and shoot tip, open and unopened flower). The heatmap function in TBtools was used for further expression analysis.

### Plant materials and treatments

Seeds of Williams 82 were sterilized using the chlorine gas method [26]. The sterilized seeds were then germinated hydroponically in a growth chamber under controlled conditions. The growth chamber provided a 16-hour light and 8-hour dark cycle, maintaining a temperature of 25 °C. The germination process continued for seven days. To investigate the effects of nitrate treatment on the seedlings, they were transferred to a modified MS (Murashige and Skoog) liquid medium [27]. This modified medium allowed for the manipulation of nitrate concentrations. The seedlings were exposed to three different nitrate concentrations: a high concentration (HN) of 54.3 mM NO_3_^-^, a suitable concentration (NN) of 18.81 mM NO_3_^-^, and a low concentration (LN) of 6.27 mM NO_3_^-^. The seedlings were maintained in these nitrate treatments for seven days.

For the drought and salt treatments, the seeds were germinated in a growth chamber under controlled conditions, including a 14-hour light period, a 10-hour dark period, a temperature of 25 °C, and a relative humidity of 60%. The resulting seedlings were then transferred to a half-strength MS solution for further growth. At the V1 stage of development, the seedlings were exposed to either 15% PEG6000 or 150 mM NaCl. These seedlings’ roots were collected 24 and 48 h after treatment. The collected roots were ground using liquid nitrogen and stored at -80 °C until further analysis.

### RNA extraction and RT-qPCR-based analysis

Total RNA was extracted from roots using the RNA Pure Plant Kit (DNase1) (Cat#CW0559S, CWBIO, Taizhou, Jiangsu, China). Agarose gel electrophoresis was employed to analyze all RNA samples, followed by quantification using a Nanodrop ND-1000 spectrophotometer V3.7.9. First-strand cDNA was synthesized using HiScripIII RT SuperMix for qPCR (Cat# R323-01, Vazyme, Nanjing, Jiangsu, China). The primers for quantitative real-time PCR (qRT-PCR) were designed using the IDT online software (https://sg.idtdna.com/) (Table S1), and their specificity was verified within Glycine max Wm82.a4.v1 genome using Phytozome BLAST. The quantitative real–time PCR was performed on a CFX96 real-time PCR system (Bio-Rad, USA) using SYBR qPCR Master Mix (Cat#Q711, Vazyme Nanjing, Jiangsu, China). The housekeeping gene *Actin11* (Glyma18g290800) was used as an internal control. Reactions were performed as follows: 95 °C for 30 s, 40 cycles of 95 °C for 10 s, and 60 °C for 30 s. The relative gene expression levels of target genes were calculated using the 2^-ΔΔCt^ comparative threshold cycle (Ct) method. The Ct values were determined from three biological replicates, with three technical replicates for each sample. Statistically significant differences in gene expression were determined using a *t*-test, * *p* < 0.05; ** *p* < 0.01, and *** *p* < 0.001.

## Results

**Identification of** ***BTB*** **gene family and prediction of subcellular localization**

We identified 122 *BTB* genes in the soybean genome through bioinformatics approaches and assigned those unique names (*GmBTB001* to *GmBTB122*) based on their chromosomal distribution and relative linear order. These genes encoded proteins ranging in length from 101 to 1020 amino acids. The molecular weights of the soybean BTB proteins varied from 11.85 to 115.16 kDa, while their theoretical isoelectric points ranged from 4.67 to 9.25. Interestingly, 90 of the identified BTB proteins were classified as unstable, with an instability coefficient exceeding 40. The remaining proteins were predicted to be stable. Subcellular localization analysis indicated that soybean BTB proteins exhibited diverse distribution patterns. They were observed in multiple cellular compartments, including the cell membrane, nucleus, and chloroplast. Specifically, 44 BTB proteins were found to be localized in both the cell membrane and nucleus. In comparison, 36 proteins were exclusively localized in the cell membrane, 31 were exclusively localized in the nucleus, and eight were located in the chloroplast and cell membrane. Notably, a subset of three BTB proteins showed specific localization within the chloroplast. The anticipated physicochemical information and subcellular localization of each GmBTB protein were presented in Table S2.

### Chromosomal location, gene duplication and Ka\Ks analyses

**Fig. 1:**
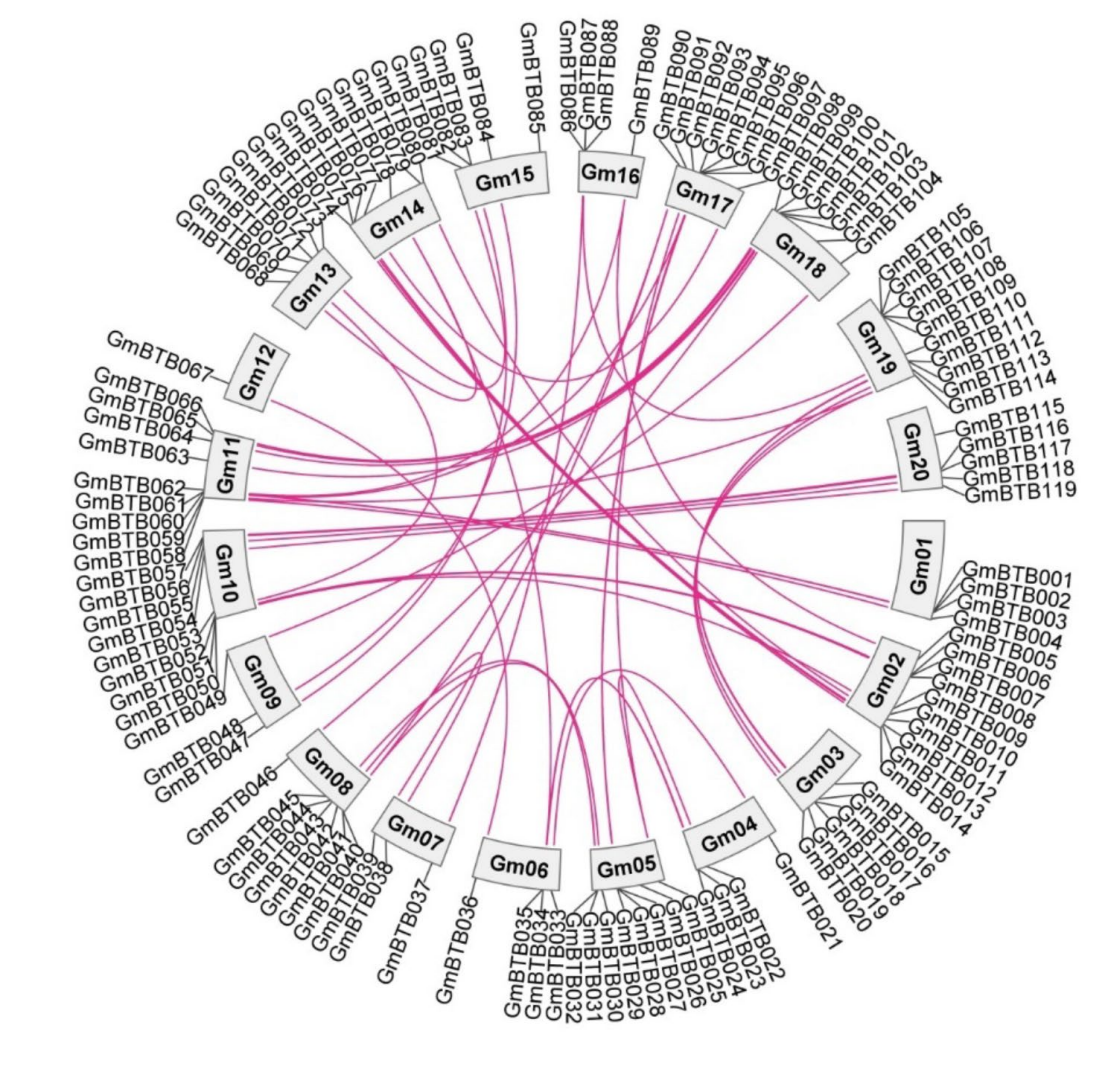
Genomic distribution and duplication of BTB genes a cross the 20 chromosomes. The pink lines represent the segmentally duplicated *GmBTB* genes

Among the *GmBTB* genes, 119 were distributed unevenly across the soybean chromosomes. Each chromosome except for chromosome 12, contained various genes, ranging from 3 to 10 (Fig. 1). Notably, chromosome 12 harbored only one gene, *GmBTB06*7. Additionally, three genes (*GmBTB120*, *GmBTB121*, and *GmBTB12*2) were identified on the unanchored scaffold_32 (Fig. S1).

To investigate gene duplication events, we examined tandem and segmental duplications among the *GmBTB* genes. Tandem duplications occur when two or more genes are located within a 200 Kb range on the same chromosome. This study identified five pairs of tandem duplicated genes (*GmBTB002/003*, *GmBTB028/029*, *GmBTB087/088*, *GmBTB093/094/095*, *GmBTB105/106*) were distributed on Chromosomes 1, 5, 16, 17, and 19, respectively (Fig. S1). Additionally, Circos analysis revealed putative segmental duplicated gene pairs among the *GmBTB* genes in the soybean genome (Fig. 1). These findings suggested that gene duplication events, including both tandem and segmental duplications, have contributed to the expansion and diversification of the *GmBTB* genes. To gain insights into the evolutionary rate of BTB domain in each gene pair, the nonsynonymous (Ka) over synonymous (Ks) ratio was calculated to assess the selection pressure during evolution. The Ka/Ks of duplicated gene pairs (either entire gene or BTB domain) were all less than 1, which tended to be pure selection, indicating that *BTB* genes sequence similarity was very high and relatively conserved during evolution as synonymous mutations are fixed more frequently than non-synonymous mutations (Table S3).

### BTB proteins classification and phylogenetic tree

**Fig. 2:**
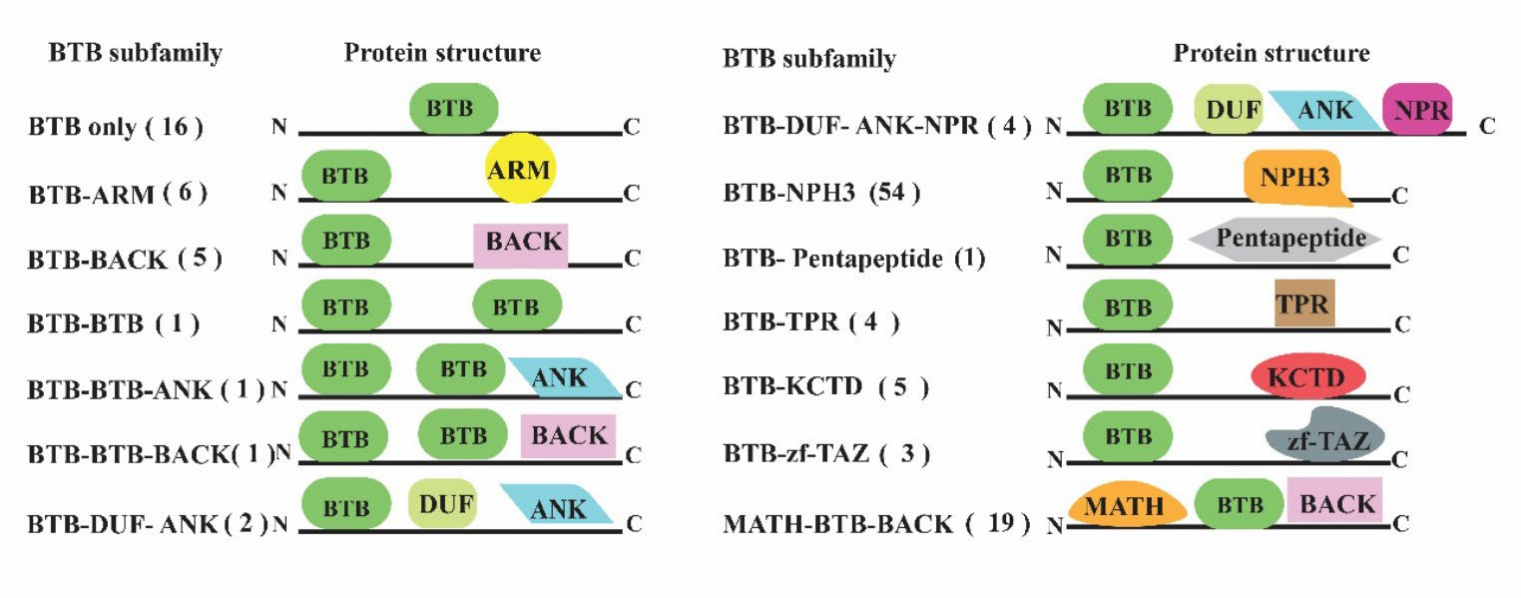
The predicted domains of BTB proteins and the number of genes in the 14 BTB subfamilies. Members of the BTB subgroup with different domains are shown. The subfamily to which the representative BTB protein belongs and the number of members in each subgroup are shown on the left and the proteins structure are shown on the right

Based on Pfam and SMART databases, all soybean BTB proteins identified in this study possessed at least one BTB domain. Most of these proteins comprised a single BTB domain, while *GmBTB80*, *GmBTB85*, and *GmBTB115* possessed two BTB domains. The distribution of these domains within the amino acid sequences exhibited a non-uniform pattern, with some domains located at the N-terminal region and others at the C-terminal region (Fig. 2). Furthermore, our analysis demonstrated that 86% of the identified BTB proteins combined with at least one additional functional domain. These domains included ARM, ANK, BTB, KCTD, BACK, DUF, MATH, Pentapeptide, NPR, TPR, and zf-TAZ. This combination of domains resulted in the formation of distinct BTB gene subfamilies, such as BTB-ARM, BTB-ANK, BTB-BACK, BTB-BTB, BTB-DUF-ANK, BTB-DUF-ANK-NPR, and MATH-BTB (Fig. 2).

**Fig. 3:**
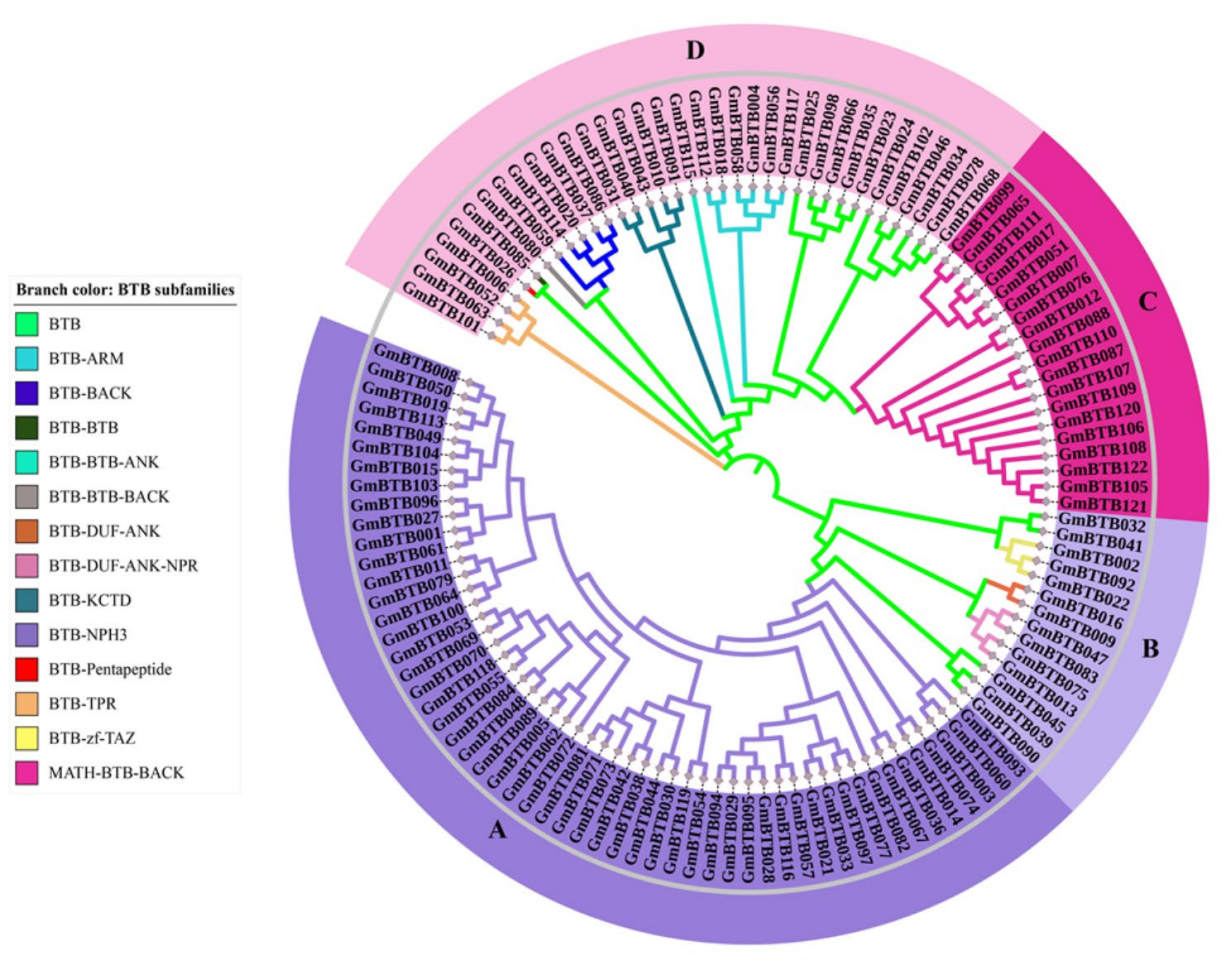
Unrooted phylogenetic tree of *BTB* genes in soybean. Deduced full-length amino acid sequences were used to construct the phylogenetic tree using MEGA 11 software by a neighbor-joining method with bootstrap replicates 1000. Based on phylogenetic tree, all 122 *GmBTB* genes were divided into four major groups, containing 14 BTB subfamilies with different domain combinations labeled by varying branch colors. A(BTB-NPH3): deep purple; B (BTB, BTB-DUF-ANK-NPR, BTB-zf-TAZ, BTB-DUF-ANK): light purple; C(MATH-BTB-BACK): light red; D (BTB, BTB-BTB-ANK, BTB-ARM, BTB-BTB, BTB-KCTD, BTB-BACK, BTB-BTB-BACK, BTB-TPR, BTB-Pentapetide): pink

In order to investigate the phylogenetic relationship and divergence of the BTB family in soybean, an unrooted phylogenetic tree was constructed using the neighbor-joining method (Fig. 3), which divided *GmBTB* genes into four major groups containing 14 BTB subfamilies, each containing 1 to 54 genes (Fig. 2). Notably, group A (BTB-NPH3 subfamily) was identified to have 54 *BTB* genes that possess the NPH3 domain. Most BTB subfamilies, such as BTB-NPH3, MATH-BTB-BACK, and BTB-ARM, were closely clustered together within the same major group. These findings were consistent across both the SMART and Pfam databases. Additionally, one new subfamily of BTB proteins was discovered, each with unique domain combinations, namely BTB-DUF-ANK-NPR and BTB-BTB-ANK. The identification of these novel BTB subgroups in soybean suggests that these genes may serve distinct functions in the growth and development of soybean.

### Gene structure and conserved motifs

**Fig. 4:**
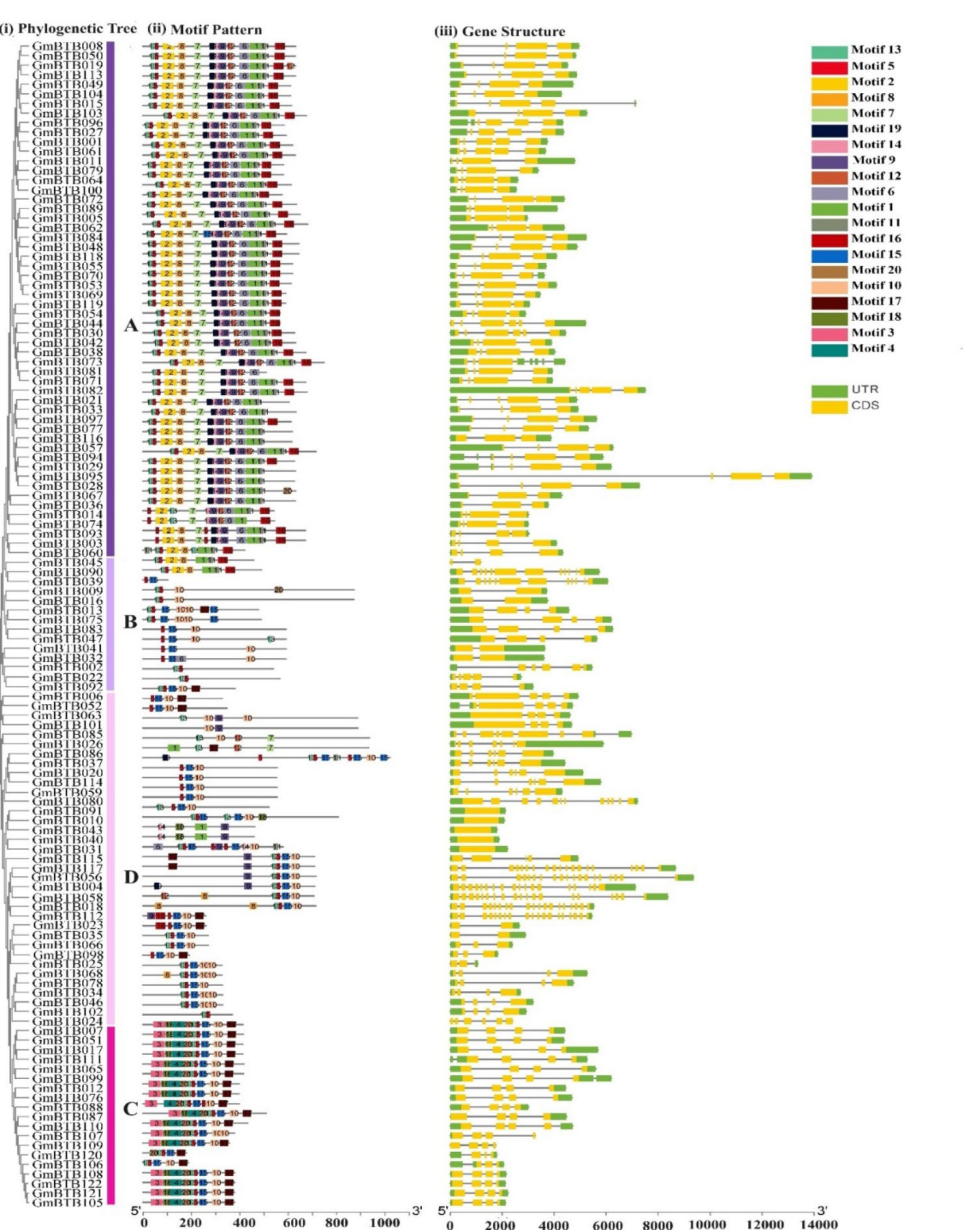
(**i**) phylogenetic relationships, (**ii**) Motif compositions gene structure of *GmBTB* genes, (**iii**) Gene structure. On the right, different colored boxes represent different motifs. The green boxes represent UTR and the yellow boxes represent exons

To investigate the diversity of motif components, we used the online MEME software to analyze the conserved motifs of 122 *GmBTB* proteins. A total of 20 distinct motifs were identified (Fig. 4ii). The result showed that motif five was found in most BTB genes, indicating that this motif may be related to the BTB domain. In addition, members of the same GmBTB groups exhibit a consistent motif composition and distribution, indicating that the GmBTB proteins in the same groups may possess similar functions. For example, proteins in group A (BTB-NPH3) possessed motifs 1, 2, and 5, while all gene members of group C (MATH-BTB-BACK) contained motifs 5 and 17. However, significant differences were also observed between different groups, with some motifs exclusively presented in specific groups. For example, motif 19 was only observed in group A and motif 3 in group C, suggesting that these motifs might have specific functions in these groups.

To gain more insight into the evolution of the soybean *BTB* gene family, we examined the exon-intron organizations of all identified *GmBTB* genes. As shown in Fig. 4iii, all *GmBTB* genes possess 0 to 18 introns. All members in group A had introns within the range of 2–5. Group B had varied number of introns ranging from 1 to 11. In addition, all group C members typically contained three introns with the exceptions of *GmBTB106* and *GmBTB120*, which contained two introns. In group D, all BTB-KCTD genes lack introns, while all BTB-ARM genes contained the highest number of introns (17 and 18 introns). Members of other subfamilies had introns ranging from 3 to 11.

### Cis‑acting elements analysis

**Fig. 5:**
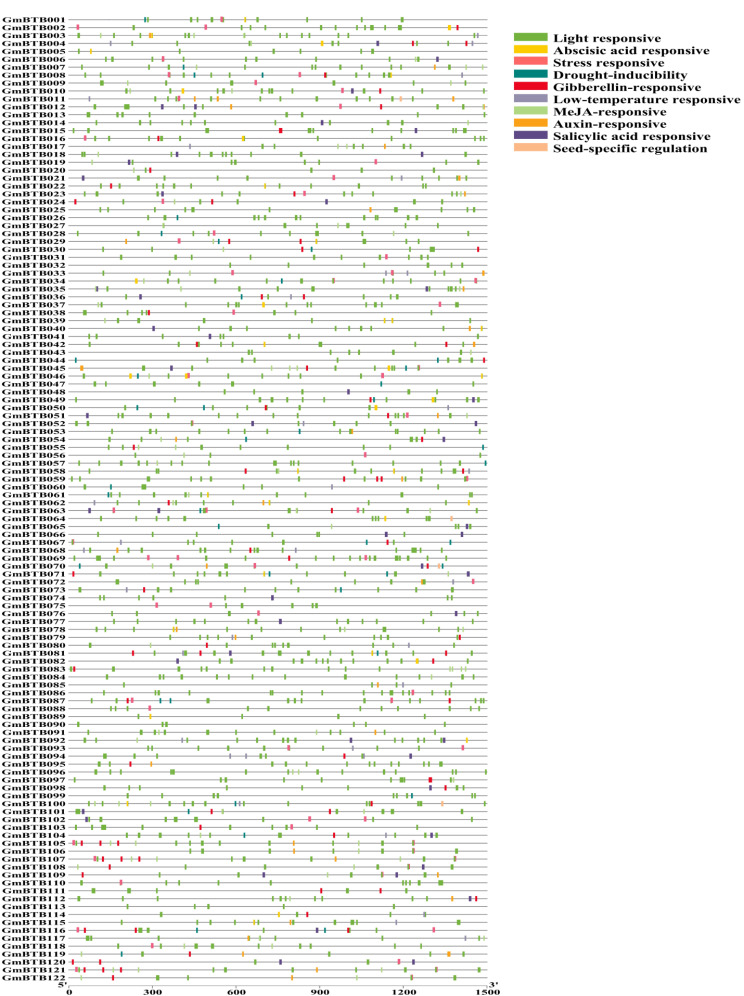
Cis-element analysis on the promoter region of the *GmBTB* genes. The potential cis-regulatory elements in the 1500 bp promoter regions were predicted by PlantCARE software. Different colors indicated the elements related to different functional categories

To identify potential regulatory elements in the promoter regions of *GmBTB* genes, the sequences located 1500-bp upstream from each gene’s protein start codons (ATG) was examined using the PlantCARE database. The analysis revealed that the identified cis-elements were mainly associated with light, hormone, and stress responses (Fig. 5 and Table S4). Among these elements, the most prevalent ones were light-responsive elements, including Box 4, G-Box, TCT-motif, ACE, ATC-motif, GATA-motif, GT1-motif, and G-Box (Fig. 5). These findings suggest that the *GmBTB* genes could respond to different stress conditions and exhibit high responsiveness to light.

### Genome synteny analysis

**Fig. 6:**
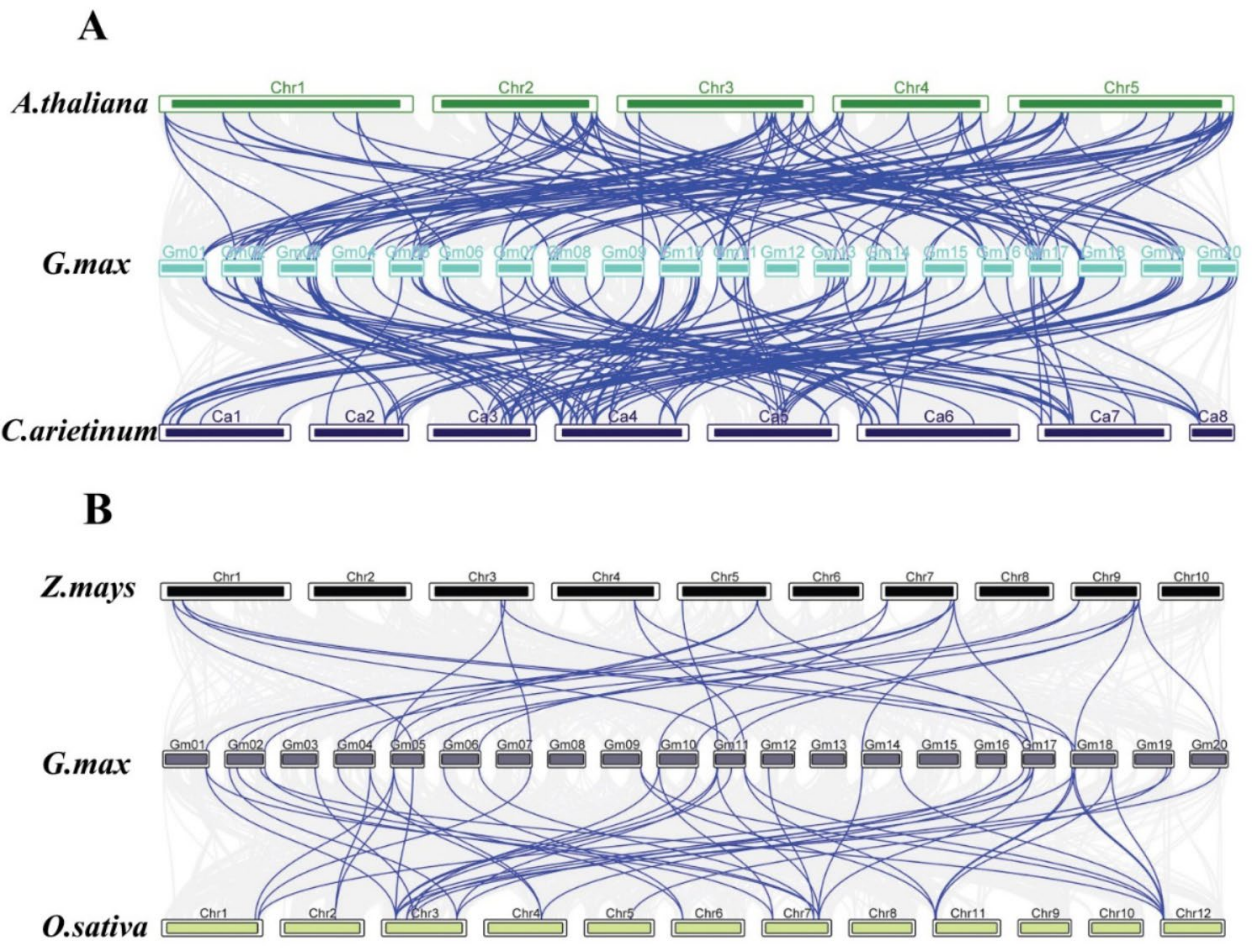
Synteny analysis of *BTB* genes between *Glycine max* and (**A**) dicotyledonous plant *Arabidopsis thaliana* and *Cicer arietinum*, (**B**) monocotyledonous plant *Zea mays* and *Oryza sativa*. Gray lines in the background indicate the collinear blocks within soybean and other plant genomes, while the blue lines highlight the syntenic BTB gene pair

To further investigate gene duplications within the *BTB* gene family, we conducted genome-to-genome synteny analysis comparing soybean with *Arabidopsis* and chickpea (both dicots) (Fig. 6A), as well as rice and maize (both monocots) (Fig. 6B). We found a higher degree of collinearity between soybean and dicots plant genomes compared to soybean and monocot. Additionally, we observed that most chickpea *BTB* genes had corresponding orthologs in soybean, with many having more than two orthologs. The presence of orthologous gene pairs suggests the conservation of gene function and potential for similar biological processes across these species.

### Gene expression pattern of the GmBTB family in soybean tissues

**Fig. 7:**
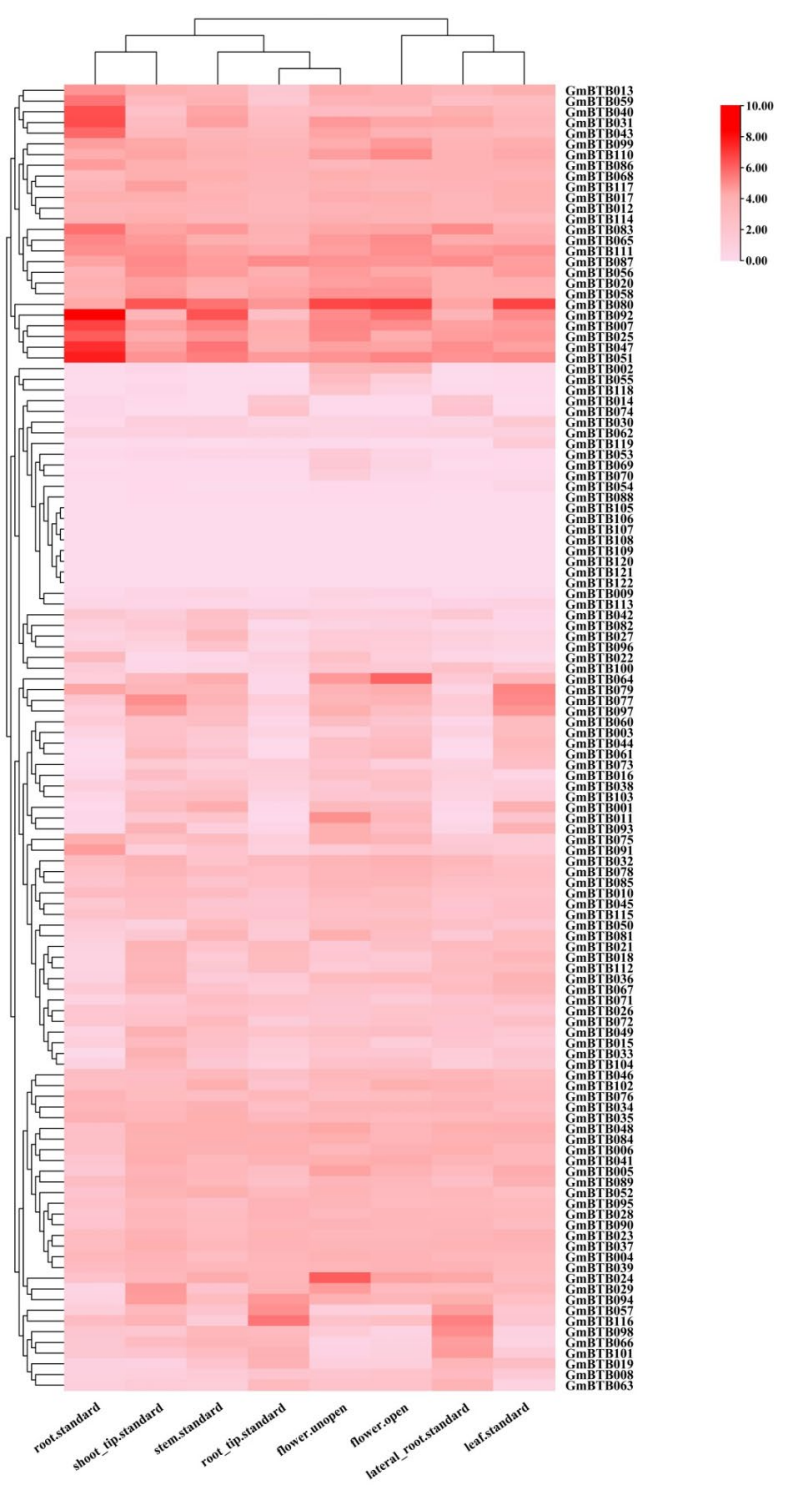
Expression patterns analysis of the *GmBTB* genes in eight soybean tissues. RNA-Seq data were used to construct the expression patterns using the FPKM values. The color scale bars on the right display the expression levels of each gene. (The level of expression was arranged horizontally)

The expression patterns (FPKM values) of all *GmBTB* genes were clustered across all tested tissues (Fig. 7). The results showed that the expression levels among *GmBTB* genes were highly varied. Among the 122 *GmBTB* genes, the expression levels were examined at least in one tissue for 114 genes. In contrast, eight genes (*GmBTB105*, *GmBTB106*, *GmBTB107*, *GmBTB108*, *GmBTB109*, *GmBTB120*, *GmBTB121*, and *GmBTB122*) were not detected in any tissue, which may be pseudogenes or had unique expression patterns not examined in our study. Some genes exhibited preferential expression across detected tissues. For instance, *GmBTB007*, *GmBTB025*, and *GmBTB051* were expressed at relatively high levels in all tissues.

Several genes showed tissue-specific expression patterns with notable levels of expression. For example, *GmBTB024* and *GmBTB064* exhibited specific high expression levels in unopened and open flowers, respectively. On the other hand, *GmBTB007*, *GmBTB031*, *GmBTB047*, and *GmBTB051* showed high expression in root tissue, indicating a potential role in root development. *GmBTB092* exhibited high expression levels in the root and stem, while lower expression levels in the shoot (Fig. 7 and Table S5). Differential expression levels of the same gene across different tissues can assist to identify a more promising candidate for in vivo studies.

### Expression pattern analysis of *GmBTB* genes under different stresses and nitrate levels

**Fig. 8:**
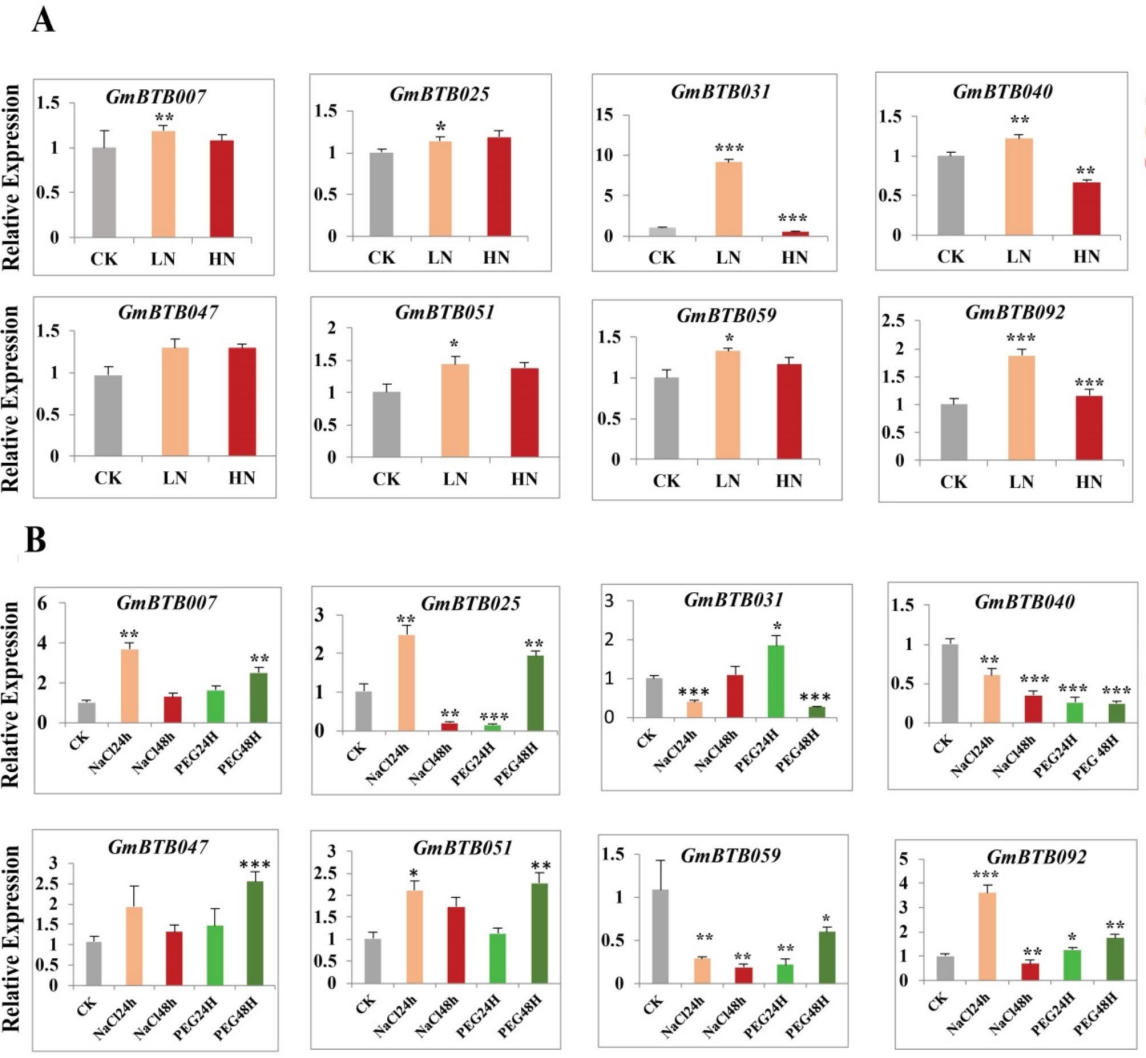
Expression profile of eight selected *GmBTB* genes in response to various stress treatments. (**A**) Under different nitrate concentrations. (**B**) Under 24 h NaCl, 48 h NaCl, 24 h PEG, and 48 h PEG treatments. The expression level was gained using qRT-PCR. Data were normalized using soybean *Actin11* (Glyma18g290800) as reference gene. Error bars indicate the standard deviation. Asterisks indicate statistically significant expression differences using t-tests (* *p <* 0.05; ** *p* < 0.01, and ***p < 0.001)

In order to conduct a more comprehensive analysis of the potential functions of the *GmBTB* gene family in soybean plants in response to various abiotic stresses, we specifically chose and evaluated the expression patterns of eight *GmBTB* genes under various nitrate levels and abiotic stresses (salt and drought) using qRT-PCR (Fig. 8A and B). The results showed that under low nitrate treatment except for *GmBTB047*, the expressions of the other seven *GmBTB* genes were significantly up-regulated, with *GmBTB031* exhibiting the highest expression, *GmBTB047* expression was not significantly up-regulated under low nitrate concentration (Fig. 8A). In other hand, all selected genes except *GmBTB031* and *GmBTB040* were up-regulated as response to high nitrate concentration treatment. *GmBTB031* and *GmBTB040* displayed a contrasting trend in response to different nitrate treatments (Fig. 8A).

As presented in Fig. 8B, the expression patterns of four genes, namely *GmBTB007*, *GmBTB025*, *GmBTB051*, and *GmBTB092*, were significantly increased after 24 h of NaCl treatment. Additionally, GmBTB047 was up-regulated, although not significantly, whereas *GmBTB031*, *GmBTB040*, and *GmBTB059* exhibited significant decreases in expression. In addition, after 48 h NaCl treatment, the expression of *GmBTB007*, *GmBTB031*, *GmBTB047*, and *GmBTB051* were up-regulated. At the same time, *GmBTB025*, *GmBTB040*, *GmBTB059*, and *GmBTB092* were significantly down-regulated (Fig. 8B). In contrast to salt stress, drought stress significantly affected the expression of the selected eight *GmBTB genes* (Fig. 8B). *GmBTB031*, *GmBTB040*, *GmBTB05*9, were significantly down-regulated while *GmBTB007*, *GmBTB025*, *GmBTB047*, *GmBTB051*, *GmBTB092* were significantly up-regulated under PEG treatment (Fig. 8B). These results indicated that BTB gene expressions are responsive to multi-abiotic stresses and different levels of nitrate.

### Gene co-expression analysis and Gene Ontology

**Fig. 9:**
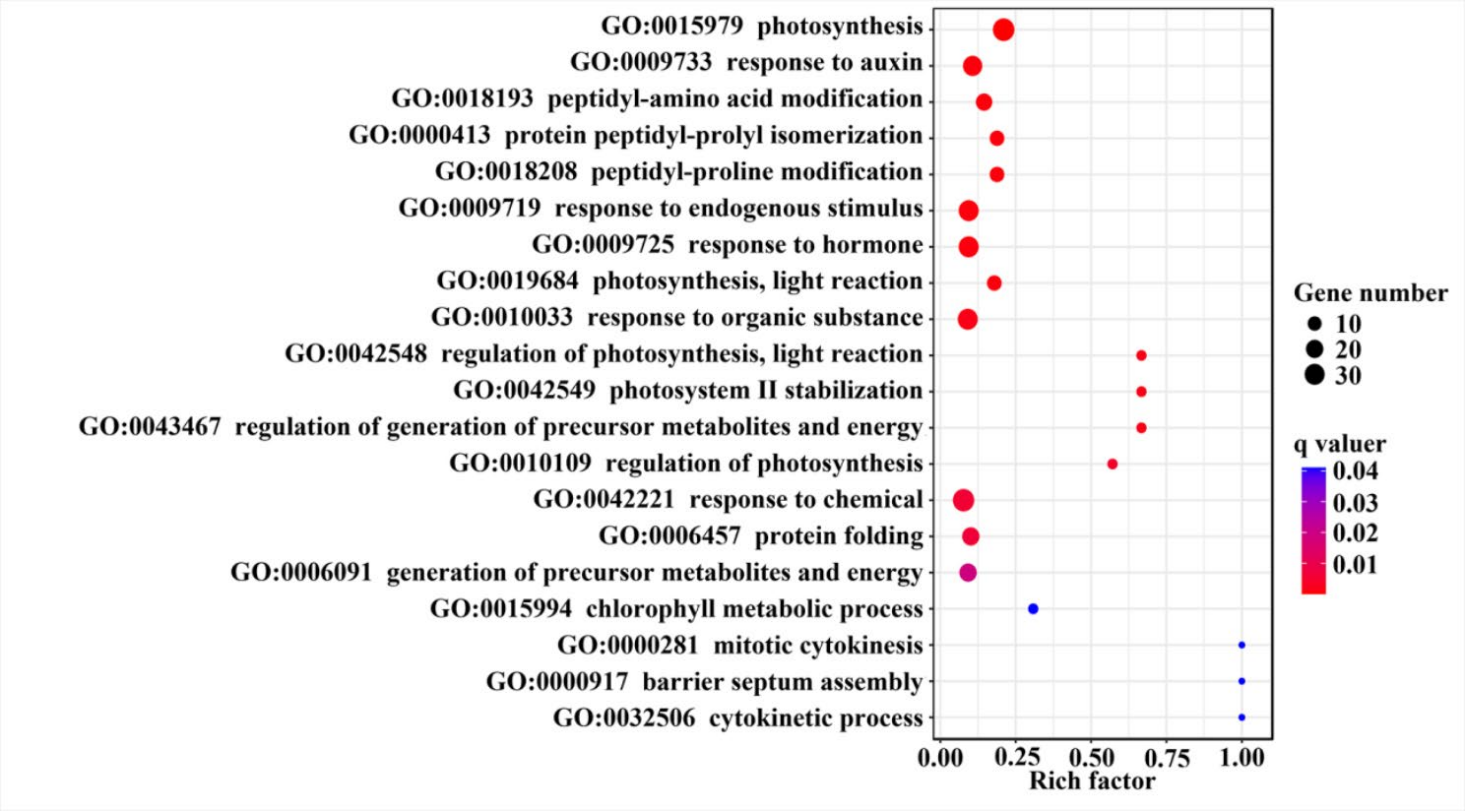
Top 20 enriched Gene Ontology of *GmBTBs* co-expressed genes. Color represents q value, and the size of the balls shows gene number

The 2053 co-expressed genes of GmBTB genes were obtained from the Phytozome database. These genes were used to identify pathways in which the *GmBTB* gene may participate using Gene Ontology enrichment analysis. The top 20 Go Ontology terms are correlated with biological processes, as shown in Fig. 9. The result revealed a significantly enrichment of gene expression related to photosynthesis related pathways (GO:0015979, GO:0019684, GO:0042548, GO:0042549 and GO:0010109), hormone response (GO:0009733 and GO:0009725), and amino acid modification or protein folding (GO:0018208, GO:0006457 and GO:0000413). Other biological process includes chemical or stimulus response (GO:0042221 and GO:0009719), protein folding (GO:0006457), metabolites and energy (GO:0043467, GO:0006091 and GO:0015994), and cytokinesis related (GO:0015994, GO:0000281 and GO:0032506). Based on these findings, *GmBTB* genes may play a crucial role in photosynthesis and hormone response, as well as their involvement in regulating critical processes associated with energy production and utilization in soybean plants.

## Discussion

BTB Domain-containing gene family has been shown to play a crucial role in regulating various biological processes, including responses to biotic and abiotic stresses [28]. Recently, extensive research has been conducted on the *BTB* genes in different plant species. However, until now, there has yet to be a genome-wide analysis of the *BTB*/*POZ* gene family in *Glycine max*. Therefore, in this study, we utilized the bioinformatics method to conduct a comprehensive and systematic analysis of the *BTB* gene family in soybean. In addition, our study investigated the expression patterns of *GmBTB* genes in response to drought, salt and different nitrate levels.

In previous studies, *BTB* gene families were identified in different plant genomes and characterized by the presence of BTB domain. These include 158 genes in rice [29], 80 in *Arabidopsis* [30], 69 in grapevine [31], 49 in sugar beet [1], and 38 in tomato [32]. Consistent with these findings, our study identified 122 *GmBTB* genes distributed irregularly on the 20 chromosomes and one scaffold. However, the differences in the number of *BTB* genes observed across diverse plant species can be attributed to variations in genome size and long-term evolutionary processes.

Through a comprehensive domain analysis, we confirmed the presence of BTB domains in all identified soybean BTB proteins. Consisting with previous studies in grapevine, tomato, and rice [29, 31, 32], we found three members of the *GmBTB* genes exhibited the presence of two BTB domains, namely *GmBTB080*,* GmBTB085*, and *GmBTB115*. Moreover, many BTB proteins exhibited additional functional domains, such as ARM, ANK, BACK, MATH, and NPH, leading to diverse BTB gene subfamilies. This result matches the BTB proteins in peach, which possess additional functional domains such as TAZ, MATH, BACK, NPH, TRP, and ANK [33].

According to the phylogenetic analysis, the GmBTB proteins were grouped into four main groups containing 14 BTB subfamilies, which shared similarities regarding their protein size, intron-exons organization, and motifs. This result suggests that the genes in similar subfamilies play a similar role in developing soybean. Similarly, in tomato, the BTB proteins were divided into four groups containing different BTB subfamilies with different domain combination, each displaying similarities in protein size and intron-exon organization [32]. Interestingly, group B contains BTB-DUF-ANK-NPR protein, a new BTB subfamily reported in peach [34], which combines the ANK, NPR, and DUF domains. ANK domains have been proven to mediate diverse protein-protein interactions [35]. In rice, proteins containing ANK participated in various physiological processes, such as responses to light treatments and NAA (1-naphthylacetic acid) [36]. NPR1 serves as a key immune regulator, playing a crucial role in systemic acquired stomatal immunity by triggering a burst of reactive oxygen species (ROS) and modulating the stomatal immune response [37]. The new subgroup BTB-DUF-ANK-NPR is proposed to potentially integrate the functions of BTB, ANK, NPR1, and DUF domains.

The subcellular localization of proteins provides insights into their functional locations. In a study by Goyal et al. [31], BTB protein prediction revealed that most BTB proteins are located in the nucleus of *V. vinifera*. Some BTB proteins were also found in the cytoplasm, plasma membrane, and chloroplast. Similarly, BTB proteins in *G.max* were predominantly located in the nucleus and cell membrane, with a small number distributed in the chloroplast. However, some difference in the subcellular localization of BTB proteins was observed between *V. vinifera* and *G.max*. These findings suggest that BTB proteins may have diverse functions in different cellular compartments. Investigating their specific localization, interactions, and functional implications in these compartments can provide a comprehensive understanding of the roles of BTB proteins in cellular processes.

Gene structures and motifs analysis provide essential information on phylogenetic relationships and are closely linked with protein function. *GmBTB* gene family members within the same group shared similar gene structures and motif compositions, suggesting they may have similar roles in plant growth and development. Our findings indicated that most *GmBTB* gene contains three introns. However, some gene family members had more introns (11 and 18). Interestingly, a few genes were also found to be intron less. Having multiple introns in a gene can delay transcriptional output, which could suppress gene expression under adverse conditions. On the other hand, genes with smaller or fewer introns may exhibit more efficient expression in response to stressful environments [38, 39]. These findings are consistent with a previous study that also reported the presence of introns ranging from 0 to 18 in BTB genes of tomato and grapevine [31].

The domains and motifs found in BTB genes are crucial for their interactions with other proteins, transcriptional activity, and DNA binding [40]. This study identified 20 conserved motifs in the *GmBTB* genes (Fig. 4). The different types and numbers of motifs in the soybean *BTB* gene may contribute to the diversity of gene functions. In addition, motif five was observed to be present in the majority of GmBTB proteins, indicating that this motif is essential for identifying the soybean *BTB* gene. Moreover, the *GmBTB* genes within the same group contained the same or similar motifs. For example, motif 2, related to NPH3 proteins, was found in all *GmBTB* genes clustered in Group A. Previous studies revealed that BTB-NPH3 plays a significant role in the plant’s response to light. It functions as a photoreceptor-interacting protein and is involved in phototropism and other light-mediated processes. Additionally, BTB-NPH3 proteins have been implicated in auxin-mediated processes, such as organogenesis and growth [9, 41]. BTB-NPH3 proteins may play a critical role in developing and adapting soybeans to the environment through their regulatory functions.

Synteny analysis could reveal the functional and evolutional connections between two species [42]. In this study, by conducting a synteny analysis of the soybean genome with four other plant genomes, we observed a notable collinearity of BTB family members between soybean and the dicots plants (*Arabidopsis* and chickpea). On the other hand, only a few BTB members exhibited collinearity between the soybean and the monocot plants (maize and rice). This finding aligns with the evolutionary relationship between dicot and monocot plants.

Plant promoters are important regulatory elements required for plant gene transcription and play important regulatory roles at the transcriptional level [43]. Light responsiveness cis-element (ACE and MRE), phytohormone regulation (MeJA, SA, and ABA) were found in grapevine and sweet potato *BTB* gene [14, 31]. Consistent with above reports, our results demonstrated that the promoters of *GmBTB* contained numerous and various cis-elements, mainly related to response to various abiotic stresses and hormone responsive, indicating that GmBTB proteins may play a role in soybean growth and response to stresses.

Gene function prediction can be initially inferred by analyzing gene expression patterns [44, 45]. Our results revealed that three genes, *GmBTB007*, *GmBTB051*, and *GmBTB080*, exhibited high expression levels across all examined soybean tissues compared with other genes (Fig. 7). This result suggests that these three *GmBTB* genes are essential in soybean development. Some specific *GmBTB* genes, such as *GmBTB007*, *GmBTB031*, *GmBTB047*, *GmBTB051*, and *GmBTB092*, displayed high expression levels in the root, indicating their potential role in root development. These findings imply the specificity of gene function during plant growth and development, aligning with previous research on the involvement of *BTB* genes in controlling root development in apple plants. [46]. Additionally, some *GmBTB* genes were expressed in various tissues, suggesting their stability and broader involvement compared to genes expressed only in specific tissues or stages of organ development [28]. In addition, through co-expression network analysis, these *BTB* genes may be involved in the biological process of hormone response and photosynthesis.

Previous studies in *Arabidopsis*, *Oryza rufipogon*, *Malus domestica*, etc. showed that BTB proteins are essential for plant response to abiotic stress and different nitrate levels [8, 13, 47]. In order to investigate the potential roles of *GmBTB* genes in responding to abiotic stresses and various levels of nitrate, we conducted qRT-PCR analysis to examine the expression patterns of eight *GmBTB* genes under drought, salt stress, and different nitrate concentrations. Therefore, these findings revealed the credibility of these genes and shed light on their potential roles in responding to salt, drought stresses, and different nitrate concentrations. It is noteworthy that all eight *GmBTB* genes responded to the treatments; however, their expression patterns exhibited variations. (Fig. 8 and Table S4). In the present study, most of the eight selected *GmBTB* genes exhibited notable regulation in response to various nitrate levels and abiotic stresses (drought and salt). Under salt and drought stresses, the expression of the most examined *GmBTB* genes was significantly up-regulated, aligning with previous studies that reported the role of pepper *CaBPM4* in enhancing plants’ tolerance to drought and salinity stresses [28]. Additionally, *OsMBTB32* transgenic plants showed significant differences in growth compared with wild-type plants under salt stress [48]. Moreover, following different nitrate concentration treatments, all selected genes except *GBTB047* were significantly up-regulated in response to low nitrate treatment. Interestingly, *GmBTB031* was significantly high up-regulated under low nitrate treatment; this result agrees with a previous study in *Arabidopsis* [11]. Uniquely, *GmBTB092* was significantly induced by both low and high-nitrate treatments. According to recent research, *MdBT2* has been found to play a crucial role in regulating the stability of the MdCIbHLH1 protein through ubiquitination. This regulation is triggered in response to nitrate levels, where low nitrate levels induce the expression of malate-associated genes, while high nitrate levels reduce their expression [12]. These findings suggest that *GmBTB* genes may interact with other genes to respond to varying nitrate levels. The results in this study demonstrate the crucial role of *GmBTB* genes in soybean responses to salt, drought, and various nitrate levels. Furthermore, it was observed that most of the selected *GmBTB* genes are involved in multiple stresses and different nitrate levels responses, suggesting the existence of cross-talk between different stress signaling pathways. However, further investigation is required to understand these candidate genes’ specific functions fully.

## Conclusions

The genome-wide study of the BTB gene in soybean has provided valuable insights into its potential functions and regulatory mechanisms. We have identified and characterized 122 BTB genes in the soybean genome through comprehensive bioinformatics analyses and expression profiling. Our study suggests that these BTB genes play essential roles in various biological processes, including plant development and stress responses. Additionally, the differential expression patterns of BTB genes under different treatments imply their involvement in soybean adaptation to abiotic and nitrate stresses. This study establishes a basis for future research on the specific functions and potential uses of BTB genes in soybean breeding and crop enhancement.

### Electronic supplementary material

Below is the link to the electronic supplementary material.

Supplementary Material 1

Supplementary Material 2

Supplementary Material 3

Supplementary Material 4

Supplementary Material 5

Supplementary Material 6

## Data Availability

All data generated or analyzed during this study are included in this published article and its supplementary information files.

## Abbreviations

Arm: Armadillo/betacatenin
ANK: Ankyrin repeats
AtBTB: Arabidopsis thaliana BTB
BTB/POZ: Bric-a-Brac/Tramtrack/Broad Complex/poxvirus and zinc finger
BACK: BTB and C-terminal Kelch
CaBPM4: Capsicum annuum L. BTB/POZ and MATH protein
GmBTB: Glycine max BTB
IbBT4: Ipomoea batatas (L.) Lam –BTB TAZ protein
KCTD: Potassium channel tetramerization domain-containing
MATH: Meprin and TRAF homology
MdCIbHLH: Malus domestica cold –induced basic helix-loop-helix
MdBT: Malus domestica BTB-TAZ
NPH3: Nonphototropic Hypocotyl3
NPR1: Nonexpressor of pathogenesis-related proteins1
NUE: Nitrogen use efficiency
OsMBTB: Oryza sativa L MATH-BTB protein
ORF: Open Reading Frame
pI: Isoelectric point
qRT-PCR: Quantitative real-time polymerase chain reaction
MeJA: Methyl Jasmonate
SA: Salicylic Acid
ABA: Abscisic Acid
ROS: Reactive oxygen species
MS: Murashige and Skoog liquid medium
