# Supplementary figures and images for: Genome-wide identification, structural and gene expression analysis of BTB gene family in soybean
Source: BMC Plant Biol. 2024 Jul 11;24:663. doi: 10.1186/s12870-024-05365-1 (PMC11238345; doi:10.1186/s12870-024-05365-1)

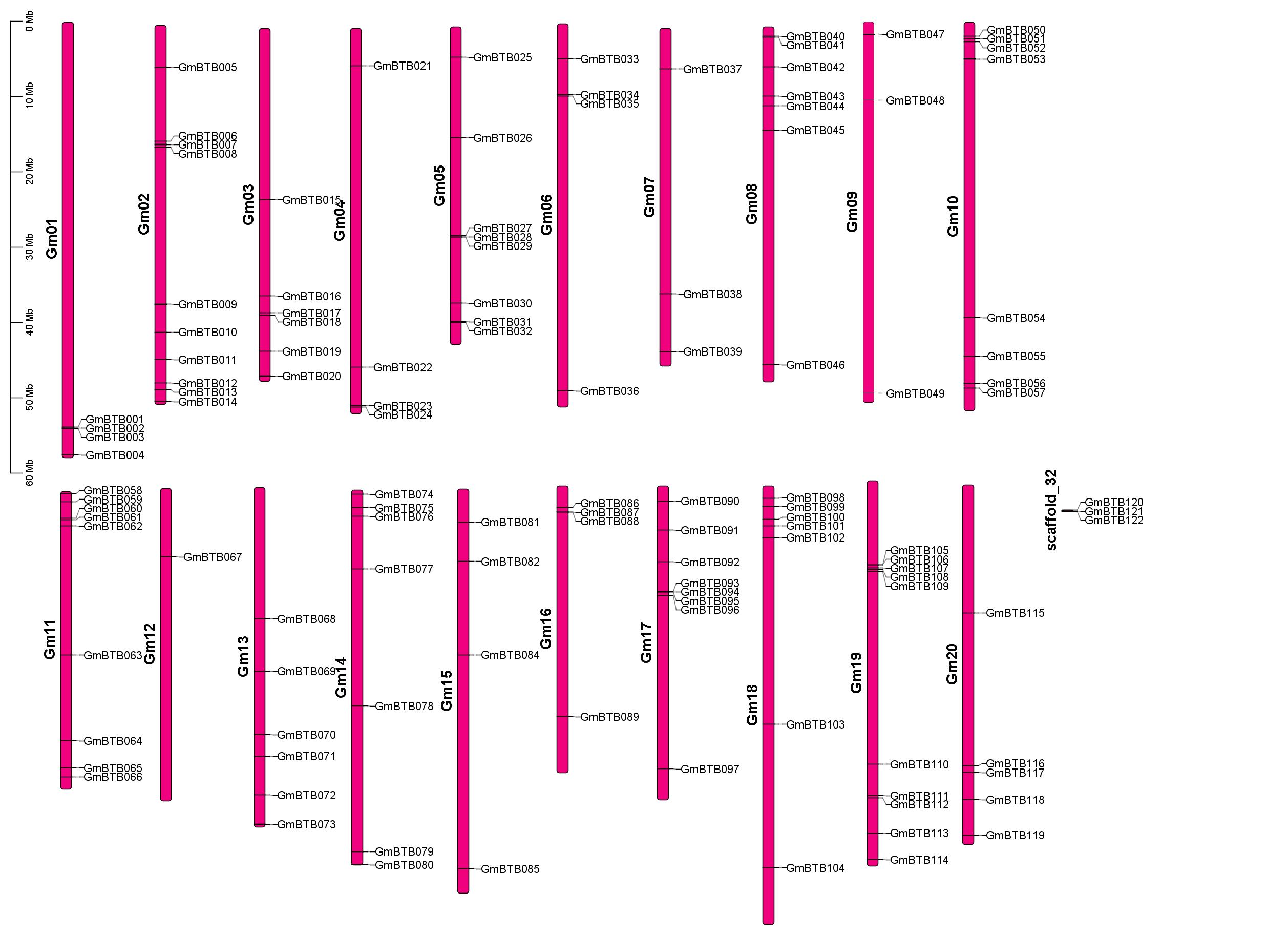

Supplement: Supplementary file 6 — Supplementary Material 6 [file 12870_2024_5365_MOESM6_ESM.jpg]
